# eGender—from e-Learning to e-Research: a web-based interactive knowledge-sharing platform for sex- and gender-specific medical education

**DOI:** 10.1186/s13293-016-0101-y

**Published:** 2016-10-14

**Authors:** Ute Seeland, Ahmad T. Nauman, Alissa Cornelis, Sabine Ludwig, Mathias Dunkel, Georgios Kararigas, Vera Regitz-Zagrosek

**Affiliations:** 1 Institute of Gender in Medicine, Charité-Universitätsmedizin Berlin, Hessische Str. 3-4, 10115 Berlin, Germany; 2 Department of Medical Education and Student Affairs, Charité-Universitätsmedizin Berlin, Berlin, Germany; 3 Structural Bioinformatics Group, Charité-Universitätsmedizin Berlin, Berlin, Germany; 4 Center for Cardiovascular Research (CCR), Charité-Universitätsmedizin Berlin, Berlin, Germany; 5 DZHK (German Centre for Cardiovascular Research) partner site Berlin, Berlin, Germany

**Keywords:** eGender, Gender medicine, Higher medical education, Sex differences, e-Learning, Knowledge-sharing platform

## Abstract

**Background:**

Sex and Gender Medicine is a novel discipline that provides equitable medical care for society and improves outcomes for both male and female patients. The integration of sex- and gender-specific knowledge into medical curricula is limited due to adequate learning material, systematic teacher training and an innovative communication strategy. We aimed at initiating an e-learning and knowledge-sharing platform for Sex and Gender Medicine, the eGender platform (http://egender.charite.de), to ensure that future doctors and health professionals will have adequate knowledge and communication skills on sex and gender differences in order to make informed decisions for their patients.

**Methods:**

The web-based eGender knowledge-sharing platform was designed to support the blended learning pedagogical teaching concept and follows the didactic concept of constructivism. Learning materials developed by Sex and Gender Medicine experts of seven universities have been used as the basis for the new *learning tools*. The content of these tools is patient-centered and provides add-on information on gender-sensitive aspects of diseases. The structural part of eGender was designed and developed using the open source e-learning platform Moodle. The eGender platform comprises an English and a German version of e-learning modules: one focusing on basic knowledge and seven on specific medical disciplines. Each module consists of several courses corresponding to a disease or symptom complex. Self-organized learning has to be managed by using different learning tools, e.g., texts and audiovisual material, tools for online communication and collaborative work.

**Results:**

More than 90 users from Europe registered for the eGender Medicine learning modules. The most frequently accessed module was “Gender Medicine—Basics” and the users favored discussion forums. These e-learning modules fulfill the quality criteria for higher education and are used within the elective Master Module “Gender Medicine—Basics” implemented into the accredited Master of Public Health at Charité—Berlin.

**Conclusions:**

The eGender platform is a flexible and user-friendly electronical knowledge-sharing platform providing evidence-based high-quality learning material used by a growing number of registered users. The eGender Medicine learning modules could be key in the reform of medical curricula to integrate Sex and Gender Medicine into the education of health professionals.

## Background

Diseases such as cancer, infections, rheumatic disease, cardiovascular disease, and diabetes show important differences between men and women. For example, the mortality of acute myocardial infarction is higher in younger women than younger men, whereas myocardial infarction generally affects men 10 years earlier than women. Cardiac diseases are more severe in men, but rheumatic diseases are more frequent in women. Men and women also differ in the predisposition for a number of cancers and infections [1–3]. Similarly, sex and gender also have an influence on pharmacodynamics and pharmacokinetics with major effects on drug therapy, ultimately resulting in under- or overdosing. Inadequate polypharmacotherapy and sex-specific drug interactions represent the majority of emergency hospitalizations in elderly women [4]. To improve health outcomes for both men and women in the future is through a more personalized medicine taking into account sex and gender differences.

Sex and Gender Medicine is a novel discipline aiming to personalize both men’s and women’s health. Sex and Gender Medicine includes biological sex with sociocultural interactions, gender, which collectively lead to differences between men and women in epidemiology, pathophysiology, manifestation, prevention, and treatment of disease.

Students of the health and medical professions need to be sensitized to sex and gender differences from the beginning of their studies of health and disease including epidemiology, pathophysiology, diagnosis, and treatment. In addition, the interaction between doctor and patient cannot be regarded in isolation from their sex and the associated genders. The teaching of Sex and Gender Medicine thus has a key role in enabling the students to recognize sex and gender differences [5]. Students should be aware of their own gender roles and existing unconscious gender stereotypes or biases affecting their activities as doctors [6].

Although several countries have already made progress with the inclusion of Sex and Gender Medicine into undergraduate medical curricula [7–10], the implementation, however, is still limited [11]. This is mainly due to the lack of adequate learning materials, systematic teacher training, and organized internet-based tools providing innovative communication strategies. Considering that knowledge is the currency of today’s economy (Communication of the European Research Area 2012, http://ec.europa.eu/euraxess/pdf/research_policies/era-communication_en.pdf) and the need to educate health and medical professionals [12], we generated a database of PubMed indexed publications screened for containing sex- and/or gender-specific analysis [13]. Subsequently, we created an innovative learning and knowledge-sharing platform of Sex and Gender Medicine, the eGender platform (http://egender.charite.de).

## Aims of the eGender platform

The overall objective of the eGender Medicine learning modules is to highlight sex and gender differences in health and disease by providing a systematic collection of evidence-based teaching material and to process these materials into innovative learning and communication tools, which aim to guarantee individual learning success. Thus, the overall aim is to educate and qualify students, physicians, and scientists in understanding the principles of Sex and Gender Medicine and to give patient-centered specific medical knowledge of sex and gender differences. Therefore, following successful completion of the modules of the eGender Medicine course, users should be able to incorporate a gender perspective from basic concepts and seven disciplines of internal medicine to their daily practice of health care. Moreover, this platform offers the chance for the Sex and Gender Medicine community to develop new fields of interest based on controversial discussions and the realization of new ideas and discoveries within scientific projects.

## Target audience

The eGender Medicine learning modules can be employed for various applications in higher education, such as the vocational training of doctors and researchers, the education of postgraduate students in modular master’s programs and as additional complementary material to seminars and lectures within curricula for medical students. Moreover, because of the flexible use of the newly developed learning tools, the course content could be of interest to other health professional groups, such as nurses and dentists, and health professional educators. The learning process and success is based on the personal “learning type,” the time that is invested, and the individual previous knowledge based on acquired educational level.

## Methods employed for the generation of the eGender platform

**Fig. 1:**
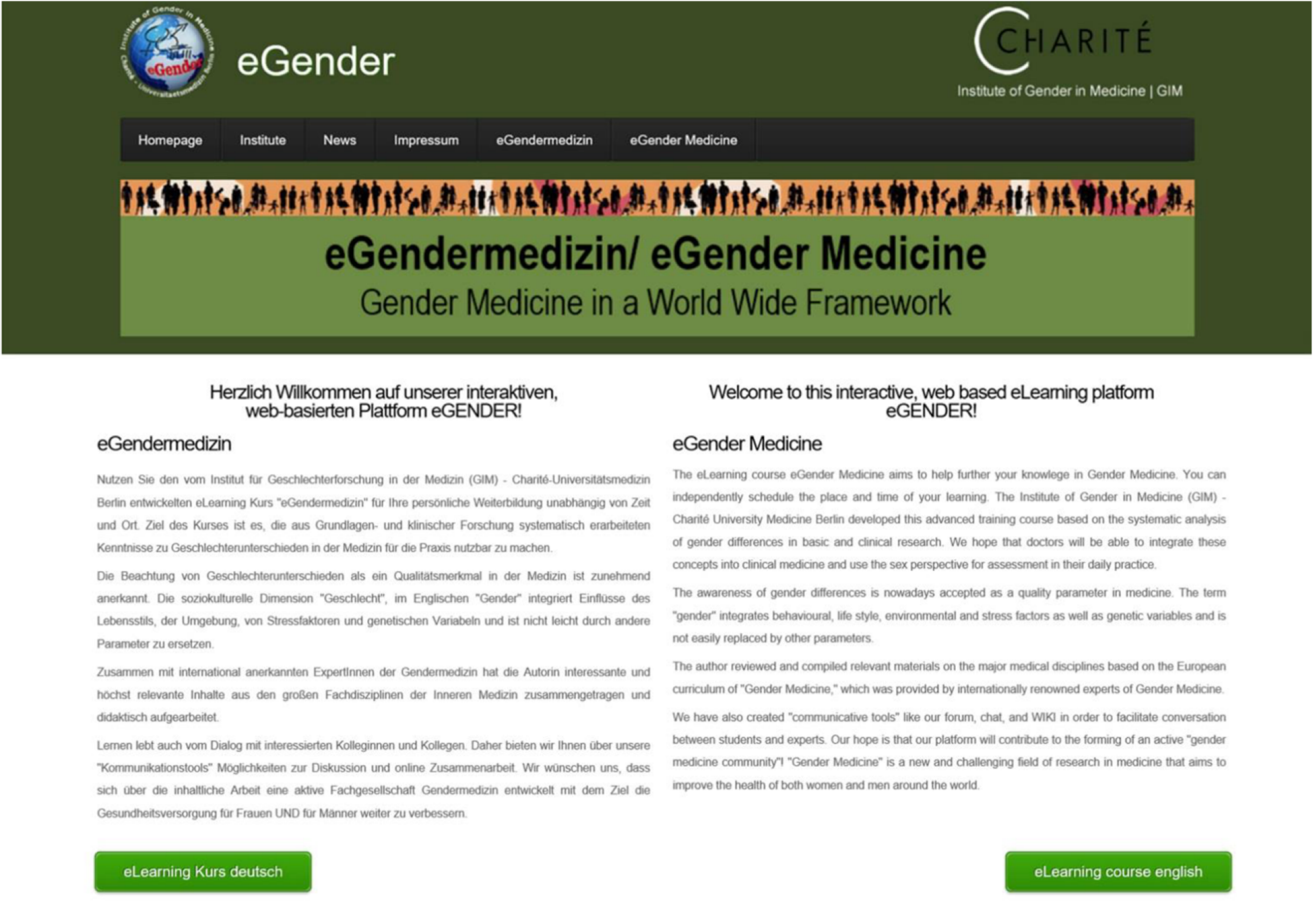
Front page eGender platform

The knowledge-sharing platform is based on the open source software Moodle. The eGender platform consists of a welcome and information page and the password-protected eGender Medicine courses in German and English language. Full access is available after online registration (Fig. 1). A personal profile including specific roles and permissions for students, mentors, and experts is provided.

The eGender platform is located on a Linux-based virtual machine in the data center of Charité-Universitätsmedizin Berlin. The website was built using PHP and JavaScript; web access is enabled via an Apache HTTP server. For optimal usage, the latest version of Mozilla Firefox, Google Chrome, or Internet Explorer is recommended. Front-end and back-end developers worked on the technical realization of the platform. This led to the creation of learning tools equipped with learning material and learning activities. The learning content and the learning objectives of the courses are based on the results of the European Gender Medicine Curriculum working group project for higher education on Master level finalized in 2011 (EUGIM Gender-Medicine, 502432-LLP-1-DE-ERASMUS-ECDEM) as well as on our own systematic analysis of sex and gender differences in preclinical and clinical science.

## The didactical and pedagogical concepts of the eGender Medicine learning modules

The eGender Medicine learning modules provide evidence-based high-quality learning and teaching material in the field of Sex and Gender Medicine using multimodality. Multimodality describes communication practices in terms of the textual, aural, linguistic, spatial, and visual resources, which are used for the development and design of the *learning tools*, provided for each disease and learning objective within the modules. This approach supports the learning process by considering the acknowledged adult learning styles. The three primary learning styles are visual, auditory, and kinesthetic and refer to how a person learns, categorizes, and processes new content. The shift from isolated text being used as the primary source of learning to audiovisual material being utilized more frequently in the digital era makes the learning process more efficient for adults. Following the andragogy principles, adults learn best in an informal situation and having roles as an active participant in the learning process. Adults will learn only what they feel they need to learn and want to know “is it relevant?” [14]. The users of eGender Medicine learning modules have the opportunity to choose from different sources of information, without having to follow a particular order of the learning tools to reach their individual learning goal. Take home massages and summaries are provided giving the opportunity to revise the new acquired knowledge or to monitor new and relevant findings. According to the user’s previous knowledge, time, motivation, and individual learning style, the most suitable *learning tool* is selected in order to reach the desired learning objectives. The e-learning tools are developed with a patient-centered, evidence-based sex and gender perspective and encompass different working materials, such as detailed learning texts, summaries, take home messages, further readings, slides, and videos. Different learning activities are also included, such as interactive tasks with respect to question quiz and term-matching games and the opportunity to listen and discuss the contributions of other experts in the field. These modal elements contribute to the user’s understanding of the specific learning issues.

The most appropriate didactic teaching concept in adult education is constructivism an experience-based theory of learning [15, 16]. This theory assumes that knowledge cannot be arranged objectively but is constructed by each person individually. Learners construct their own understanding and knowledge of the world through experiencing things and reflecting those experiences. It is a learning process which allows the users of the eGender Medicine learning modules to actively shape their learning environment based on reliable and trust-worthy sources and thus independently of their knowledge. The learners of eGender Medicine modules will be able to combine new information from the learning environment with their previous knowledge in order to advance their own understanding. If the new information, however, disagrees with the previous knowledge or experience and reconciliation of the two is not possible, it will be necessary to change the knowledge structure such that the new information will again become meaningful to the learner.

**Fig. 2:**
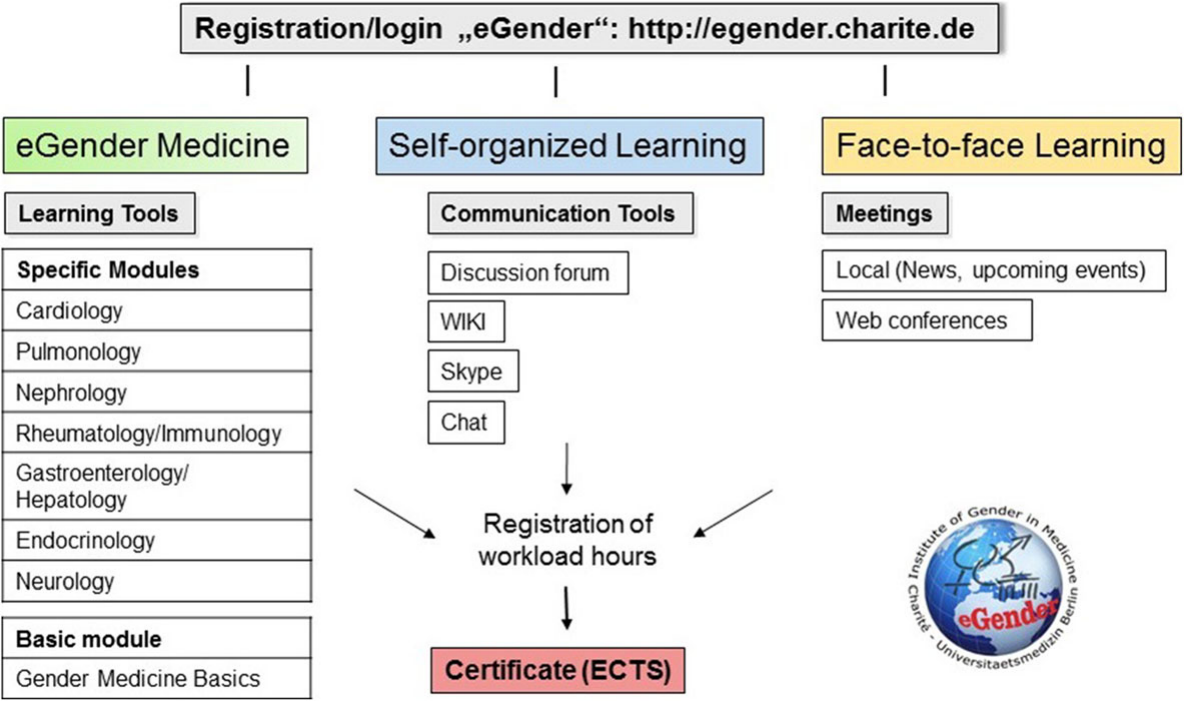
Blended learning in Sex and Gender Medicine

The pedagogical concept of *blended learning* is a successful strategy combining e-learning with face-to-face learning and online collaboration. Blended learning is facilitated by the effective combination of different modes of delivery, models of teaching, and styles of learning and is based on transparent communication of all parties involved within a module (Fig. 2). The eGender platform supports the concept of blended learning. All subjects are taught following the concept of a spiral curriculum and taking into account the three pillars of the blended learning concept. Increasing complex learning contents are offered in the e-learning modules, in the lectures, in online collaboration tasks as well as tasks and teaching formats aiming at the practical transfer of the acquired knowledge.

The eGender platform offers a wide range of learning contents and effective learning methods for providers of accredited vocational trainings in the field of Gender Medicine on Master level as well as for students in order to fulfill the Bologna criteria and obtain the required European Credit Transfer and Accumulation System (ECTS) points. The eGender learning modules can be easily integrated into such vocational trainings and master modules with minor adjustments according to the national and local needs.

1. e-Learning: The course completion status gives a direct feedback to the students on the current status of their work, their individual workload hours, and tasks that they still have to effectuate.
2. Online collaboration: The use of communication tools like chats, discussion forums, Skype, and Wiki being offered on each site of the different learning modules provide the opportunity to collaborate with each other, allow teachers/mentors to monitor, and check the work of the students, to provide tasks for the *take home exams* and monitor the time needed and the results achieved.
3. Face-to-face learning: The eGender platform offers the possibility to announce symposia and congresses as “upcoming events” or “latest news.” Therefore, students can be informed on upcoming events close to their place of residence. Most providers offer trainings of 3–5 days including lectures and seminars.

## Structure and usage of the eGender Medicine learning modules

The eGender Medicine learning modules consist of 28 different courses (diseases or complexes of symptoms), which are thematically associated to eight modules: Gender Medicine basics, cardiology, pulmonology, nephrology, rheumatology, gastroenterology/hepatology, endocrinology, and neurology. Epidemiological and pharmacological facts are represented within each discipline. All modules and courses are structured in the same way. The main part covering the evidence-based knowledge material are the seven *learning tools* offered for each course. Learning goals and learning content for the eGender Medicine learning modules build on the European Gender Medicine Curriculum for higher education. This material is updated every year and new diseases and disease patterns are added. Three main widgets are provided for each course: (1) learning tools, (2) communication tools, and (3) LINKs to international Gender Medicine communities and databases providing publications in Gender Medicine, e.g., GenderMedDB (Table 1).

**Table 1:** List of eGender Medicine module and course titles

Module	Course	Key terms
Module 1: Gender Medicine—Basics	1.1 Gender Medicine—Basics	Sex, gender, personalized medicine, gender equality, social determinants of health
Module 2: Gender differences in cardiology	2.1 Cardiovascular risk factors—traditional and new!	Risk factors, pregnancy, autoimmune diseases, Mosca Score, prevention
	2.2 Heart failure diseases	Heart failure with preserved (normal) ejection fraction (HFPEF) or diastolic heart failure, Cardiomyopathies
	2.3 Chronic ischemic heart disease	Chronic ischemic heart disease, pathophysiology
	2.4 Acute coronary syndrome/myocardial infarction	Risk factors, non-obstructive coronary artery disease, symptoms, outcome
Module 3: Pulmonology	3.1 Allergic airway diseases	Asthma, allergic airway disease, environmental factors, genetic predisposition, dyspnea
	3.2 COPD and female smokers	COPD, smoking, shortness of breath, corticosteroids, BODE index, spirometry
	3.3 Lung cancer	Cancer, smoking, CT, estrogen receptor, fatigue
	3.4 Sleep disturbances	Sleep, fatigue, insomnia, obstructive sleep apnea, restless legs, snoring
Module 4: Renal diseases	4.1 Pathophysiology	Endocrine function, testosterone deficiency, uremic state, peritoneal sclerosis
	4.2 Clinics and diagnosis	Chronic renal disease, diabetic nephropathy, albuminuria, diabetes mellitus, polycystic kidney disease
	4.3. End-stage renal disease	ESRD, transplantation, dialysis, age, diabetes
Module 5: Rheumatology	5.1 The immune system	Autoimmunity, humoral- and cell-mediated immunity, antibodies, lymphocytes, cytokines, microchimerism
	5.2. Systemic lupus erythematosus	Autoimmune, inflammatory, renal disease, hypertension, young women, Klinefelter’s syndrome
	5.3 Multiple sclerosis	Central nervous system, autoreactive CD4+ T cells, demyelisation, prolactin
	5.4 Rheumatoid arthritis	Inflammation, auto-antibodies, smoking, pregnancy, Anti-TNFα medications
Module 6: Gastroenterology/hepatology	6.1 Autoimmune diseases	Autoimmune hepatitis, primary biliary cirrhosis, primary sclerosing cholangitis, fetal microchimerism, cirrhosis, fatigue, anorexia, amenorrhea
	6.2 Infectious diseases	Hepatitis B virus, hepatitis C virus, infection, HBsAg seroconversion, hepatocellular carcinoma, liver fibrosis, viral genotype
	6.3 Mechanical diseases	Erosive reflux disease, non-erosive reflux disease, lower esophageal sphincter, hiatal hernias, heartburn, Barrett’s esophagus
	6.4 Functional diseases	Abdominal pain, psychological distress, widespread gastrointestinal disorder
	6.5 Multifactorial diseases	Ulcerative colitis, Crohn’s disease, inflammation, X chromosome, menstrual cycle, pregnancy,immunosuppression
Module 7: Endocrinology	7.1 Insulin resistance and beta-cell secretion	Pre-diabetes, diabetes mell., obesity, OGTT, IGT, PCOS
	7.2 Appetite and weight gain	Obesity, lifestyle, disease, stress, puberty, adiponectin, energy metabolism, BMI
	7.3 Bone structure and osteoporosis	Primary osteoporosis, secondary osteoporosis, osteoporotic fractures, periosteal growth, bone mineral density, menopause
Module 8: Neurology	8.1 Inflammatory neurological diseases	multiple sclerosis, amyotrophic lateral sclerosis, demyelination of neurons, central nervous system, inflammation, autoreactive CD4+ T cells, autoimmune diseases, TNFalpha,interferon
	8.2 Degenerative neurological diseases	Alzheimer’s disease, Parkinson’s disease, dementia, agitation, depression, strogen, ß-amyloid plaque, brain function, dopamine, rigidity, tremor, functional disability
	8.3 Epilepsy	Seizures, catamenial epilepsy, teratogenic effect of antiepileptic drugs
	8.4 Stroke	Cardio-embolic, atherosclerotic and lacunar stroke, atrial fibrillation, hypertension

The modular structure of the eGender Medicine courses follows the Bologna criteria with the aim to harmonize the curriculum design all over Europe and to fulfill the criteria for the certification to allocate internationally recognized ECTS points. The widget *course completion status* shows the requirements and the completion status progress. Registration of workload hours is crucial in order to receive a certificate of attendance for the e-learning courses and the opportunity to apply for ECTS points at the local universities and institutions. It is a common practice to assign ECTS points for higher education and qualifications, which has been completed by an exam.

To raise gender awareness and sensitize users without prior knowledge, the module on *Gender Basics* offers an introduction to the topic. For advanced users, more discipline-focused modules will increase the user’s knowledge on practical aspects of Sex and Gender Medicine and strengthen their ability to use the gender perspective as an assessment and behavioral tool in their daily practice. The stage of knowledge building should always be followed by a section of interaction or discussion to reinforce and expand the students’ understanding of the topic with the input of different professional but also regional backgrounds.

Users are encouraged to interact and work together by using the discussion forum, the Wiki and the chat. The communication tools also give room for mentoring and support by peers, mentors, and experts. The participation in a discussion and the presentation of a group’s findings using the Wiki tool allows for the evaluation of the learning progress of each user.

## Success of the eGender Medicine learning modules and proof-of-concept

**Fig. 3:**
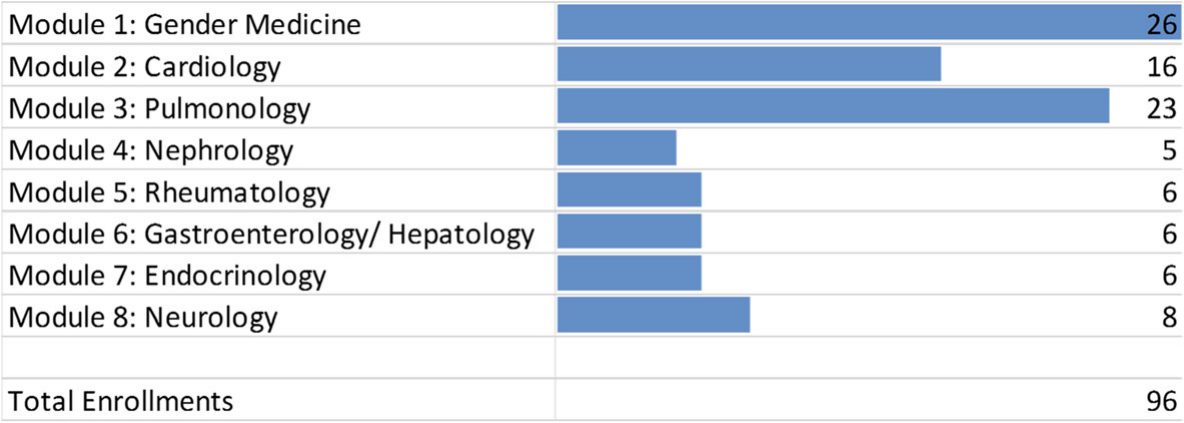
Total enrollments in eGender medicine learning modules

Statistical analysis of the users’ access data was performed since the test period in January 2014. The server logs collect the information required for establishing the connection, such as the name of the retrieved file, the date and time of retrieval, the amount of data transferred, the web browser, and the requesting domain. These data are statistically evaluated in order to optimize the technical quality and to get information about the popularity ranking of the webpage. Google’s analytics showed 3858 visitors that have had at least one session within the selected date range January 2015–December 2015 where a session is the period time a user is actively engaged with the website. The most sessions were reported from the USA, Germany, and Japan with a growing number of users from Germany, Austria, Italy, Sweden, and Pakistan. The most frequently accessed module was found to be “Gender Medicine—Basics” (Fig. 3), and the registered participants favored the discussion forum over other available communication tools.

Proof-of-concept is the implementation of a Master Module “Gender Medicine—Basics” into the Master Program Public Health (MPH) at the Charité-Universitätsmedizin Berlin. Admission requirements were a bachelor’s degree or minimum of 3-years medical studies or applicants with an equivalent degree of third level education programs, e.g., pharmacology and nursery. The Master Module addressed medical doctors, social scientists in the medical field, health care professionals, and pharmacologists.

The Master Module aimed at teaching the fundamental principles and scientific standards of Sex and Gender Medicine in selected medical disciplines: introduction in gender medicine, methodology of gender-specific research, cardiology, endocrinology, neurology, pulmonology, pharmacology, psychosocial aspects, prevention, and men’s health.

The module addressed the learning objectives and enabled the students:

1. To write a succinct and concise analysis of case histories including gender-specific aspects of medical diagnosis and treatment
2. To recognize that gender determines the outcome of numerous widespread diseases and influences therapy, medication, prevention, and rehabilitation
3. To explain gender and sex differences in diagnoses and therapy and illustrated these with examples
4. To perform online literature research with regard to gender-specific medical questions
5. To improve their communication skills, combine and interpret evidence-based medical knowledge, and argue in peer groups in favor of the necessary medical approach and treatment

In 2011, 14 participants with different academic and professional background attended the Master Module. They were asked to fill in a questionnaire with 25 questions focusing on the learning progress, course content, learning materials, facilities, assessment, and course organization. The questionnaire contained questions with a Likert scale (do not agree = 1 to strongly agree = 3) and open questions. Seven participants (out of 14) returned the questionnaire. The participants considered their achieved learning progress as having fulfilled their expectations (45 % “agree” and 55 % “strongly agree”). Twenty-nine percent considered the assessment within the module as “good” and 71 % as “excellent.” One participant replied, “I found the program to be very organized; there was a course outline of the key points, as well as notes on expected outcomes.”

In 2012, the same questionnaire was used. Ten were returned (out of 25). Fifty percent of the participants agreed that the objectives of the modules were clearly defined, and 100 % thought that the module improved their academic skills (80 % “agree,” 20 % “strongly agree”). Forty percent agreed that the module and its content are useful for their work as physician/medical specialist, and 20 % strongly agreed to this.

In 2013, the vocational training for Gender Medicine was evaluated with nine questions using the Likert scale from 1 = “excellent” to 6 = “insufficient” and four open questions. The questions were covering aspects like the overall quality of the module, course content, usability, selected teachers, organization, networking possibilities, and facilities. Seven questionnaires were returned (out of 15). The mean score of the overall quality of the module was 1.4 and 1.6 of the course content, its usefulness and transfer to the professional context was rated with a mean score of 1.3, achieved expectations with a mean score of 1.9, and planning and organization with a mean score of 1.1. One participant stated “Great padagogical concept, very good material, excellent management, I had the feeling that every minute of each lecture was important and useful for me”.

## Discussion

The eGender platform is a novel and innovative knowledge-sharing platform important for sex and gender medical education. The eGender Medicine learning modules contribute to excellence in health professions education, fostering innovation in the field of Sex and Gender Medicine and research, ultimately enhancing sustainability for knowledge transfer and incentives for new research projects. It makes education for health-related and medical professionals and researchers more responsive to social needs. This approach supports the implementation of Sex and Gender Medicine knowledge into health professions education and research and fosters personalization in medicine aiming at improving the health status of men and women worldwide.

e-Learning is very flexible in contrast to printed books—it is dynamic, changing, and adapting itself to new social situations, new technologies and new forms of learning [17]. This concept is of special importance for a new discipline like the Sex and Gender Medicine, with an extensive discussion about the next steps to develop more effective methods and statistical analysis approaches, which could be used for answering sex- and gender-sensitive basic research questions to improve the evidence-based data of the impact of sex and gender on diseases and on prevention measures. As an educational tool, the eGender platform is suitable to be used as a complementary self-study tool or as a stand-alone tool for courses or workshops on Sex and Gender Medicine.

Barriers for the e-learning approach could be that students and institutions are often not sufficiently prepared for the choices that present themselves in an e-learning context. This leads to an overtaxing of students and teachers especially in the beginning. This can be solved by educating students and experts for mentoring and providing a helpdesk for any questions concerning the use of the platform. Another barrier might be to develop didactic material for different target groups. This barrier will be overcome by involving experts in the field of didactics and front-end developers to develop new user-friendly learning environment and by conducting expert discussions among all stakeholders.

The need to implement sex and gender aspects into medical curricula as a compulsory part of the studies requires cultural and conceptual changes. Simunovic, V. J. et al. [18] assessed attitudes towards curriculum reforms in different academic, economic, and social environments among 776 teachers from two Western European medical schools, i.e., Belgium and Denmark, and seven medical schools in three countries in post-communist transition, i.e., Croatia, Slovenia, Bosnia, and Herzegovina. The data showed that teaching staff from medical schools in Bosnia and Herzegovina had a more positive attitude towards reforms of the medical curriculum than those from medical schools in Croatia or Slovenia or Western Europe. Significant predictors of positive attitudes towards medical curriculum reform in post-communist transition countries, but not in Western European schools, were younger age and female gender in Bosnia and Herzegovina. These aspects have to be considered when thinking about a time-frame for integrating Sex and Gender Medicine curriculum globally. The feasibility of curriculum reforms has already been shown by several universities, such as Charité- Universitätsmedizin Berlin [19], Radbound University Nijmegen in the Netherlands [7], Karolinska Institute in Sweden [20], several universities in Austria [8], Italy, Canada [10], and the USA [21]. Usage of the eGender Medicine learning modules could help to accelerate this process.

## Conclusions

The eGender Medicine learning modules provide a pedagogically justified and appropriate opportunity to disseminate evidence-based sex- and gender-sensitive knowledge in medicine. It helps to implement Sex and Gender Medicine into health professions education with the aim to get a prominent place of sex and gender aspects in medical curricula and innovative research. Access to knowledge in a timely efficient and economical manner and the option to communicate sufficiently within the Sex and Gender Medicine community will foster awareness of sex- and gender-responsible research, science, and medical care.

## Abbreviations

ECTS: European Credit Transfer and Accumulation System
eGender: Electronical Gender Medicine platform
eGender Medicine: Electronical Gender Medicine learning modules
SOL: Self-organized learning
